# Hemostatic Evaluation With Viscoelastic Coagulation Monitor: A Nicu Experience

**DOI:** 10.3389/fped.2022.910646

**Published:** 2022-05-10

**Authors:** Giacomo Simeone Amelio, Genny Raffaeli, Ilaria Amodeo, Silvia Gulden, Valeria Cortesi, Francesca Manzoni, Nicola Pesenti, Stefano Ghirardello, Fabio Mosca, Giacomo Cavallaro

**Affiliations:** ^1^Neonatal Intensive Care Unit, Fondazione IRCCS Ca' Granda Ospedale Maggiore Policlinico, Milan, Italy; ^2^Department of Clinical Sciences and Community Health, Università Degli Studi di Milano, Milan, Italy; ^3^Division of Biostatistics, Department of Statistics and Quantitative Methods, Epidemiology, and Public Health, University of Milano-Bicocca, Milan, Italy; ^4^Neonatal Intensive Care Unit, Fondazione IRCCS Policlinico San Matteo, Pavia, Italy

**Keywords:** thromboelastography, hemostasis, hemorrhage, VCM, newborn

## Abstract

**Background:**

Viscoelastic coagulation tests provide valuable information in neonatal intensive care units (NICUs), but the lack of reference intervals still limits their decision-making power according to gestational age. The aim of the present study is to evaluate the hemostasis of a cohort of full-term (FT) and late-preterm (LP) infants using the viscoelastic coagulation monitor (VCM®) system, a new portable device that uses untreated whole blood.

**Methods:**

An observational study was performed to identify non-coagulopathic FT and LP infants admitted to III° level NICU (January 2020 to December 2021) with a VCM test in the first 72 h of life.

**Results:**

Forty-five patients were enrolled, 26 FT and 19 LP. No statistical differences in hemostatic parameters were observed between FT and LP nor between stable and unstable neonates. Clotting time (CT) was positive correlated with PT (*p* = 0.032), not with aPTT (*p* = 0.185). From linear regression, platelet resulted associated with: clot formation time (CTF, *p* = 0.003), alpha angle (Alpha, *p* = 0.010), amplitude at 10 (A10, *p* = 0.001), amplitude at 20 min (A20, *p* < 0.001), maximum clot firmness (MCF, *p* < 0.001); and fibrinogen was associated with: A10 (*p* = 0.008), A20 (*p* = 0.015) and MCF (*p* = 0.024). Compared to the adult reference population, neonates showed shorter CT (mean (SD): 5.3 (1.4) vs. 7.0 (0.9) min, *p* < 0.001), CFT (2.4 (0.7) vs. 2.8 (0.6) minutes, *p* < 0.001) and higher Alpha (60.8 (6.3) vs. 55 (5)°, *p* < 0.001). In addition, the neonatal cohort showed an early transient difference in amplitude and fibrinolysis, as follows: A10 (28.0 (5.0) vs. 26 (4) VCM units, p =0.004), A20 (34.8 (5.0) vs. 33 (4) VCM units, p =0.012), and LI30 (99.8 (0.5) vs. 99 (1)%, p <0.001).

**Conclusions:**

The viscoelastic profile of FT and LP infants assessed with VCM showed a hemostatic competence characterized by accelerated coagulation and clot formation time, in line with other viscoelastic techniques. VCM system provides promising applications in the NICU setting.

## Introduction

Viscoelastic coagulation tests help evaluate and manage coagulation issues in neonatal intensive care units (NICUs) (1–7). While the hemostatic balance is maintained, pro- and anti-coagulant factors are reduced in newborns (8). Consequently, the alterations of conventional exams, such as prothrombin (PT) and activated partial thromboplastin time (aPTT), are not strictly related to an increased hemorrhagic risk (9–11). A viscoelastic evaluation provides a global and dynamic analysis of hemostasis, from the formation of the clot to the lysis, taking into account both plasmatic and cellular drivers of the clotting process. As the *in-vivo* hemostasis is thoroughly evaluated, viscoelastic tests may allow targeted trans fusional and pharmacological therapy.

Furthermore, this technique guarantees a rapid assessment with low blood volumes within short turnaround times, which is essential in an intensive neonatal setting. The main obstacle to the viscoelastic tests spreading in NICUs is establishing physiological reference ranges in neonates for the different devices. Indeed, clotting competence is dynamic, strictly connected to fetal and postnatal maturation, presenting differences according to gestational age (GA) and postnatal age (12, 13). Thromboelastography (TEG) and rotational thromboelastometry (ROTEM) are the widest known and commonly used methods in clinical practice, but currently, a novel device that uses untreated native blood, named Viscoelastic Coagulation Monitor (VCM®), is available. However, the experience in neonatology with this new application is scanty. The increase of knowledge about neonatal viscoelastic properties is key to promoting the viscoelastic tests as decision-making contributors for transfusion practices in NICUs. Hence, we designed an observational study with the primary objective of describing the VCM profile in a cohort of full-term (FT) and late preterm (LP) non-coagulopathic newborns admitted to the NICU of our center in the first 72 hours of life. Secondary objectives were to (1) study the relationship between the main VCM parameters with conventional laboratory tests; (2) investigate differences between the reference values of VCM in our cohort compared to the adult population.

## Materials and Methods

This observational study was conducted in the III° level NICU of Fondazione IRCCS Ca' Granda Ospedale Maggiore Policlinico, Milan, Italy. The study was approved by the local ethics committee (Milan Area 2, Italy) with approval number 1258_2021.

### Study Population

Neonates admitted to the NICU were assessed for eligibility between January 1, 2020, and December 31, 2021, with a GA ≥34 weeks and a VCM test registered in the first 72 h of life. Newborns suffering from conditions with a potential hemostatic impact (spontaneous bleeding, perinatal asphyxia in hypothermia, early-onset sepsis, thrombocytopenia, severe anemia) were excluded from the analysis. Thrombocytopenia was defined as a platelet count <130^*^10^3^/μL; severe anemia was defined as Hb <9 g/dL. All patients exposed to any blood products before the VCM test were excluded. VCM parameters, complete blood count, standard coagulation tests (PT, aPTT, and Fibrinogen), and venous pH were collected for each patient. For patients with more measurements available, only the first was considered. In addition, GA, weight, gender, obstetric, and perinatal anamnesis were recorded.

### Viscoelastic Coagulation Monitor Parameters

**Figure 1:**
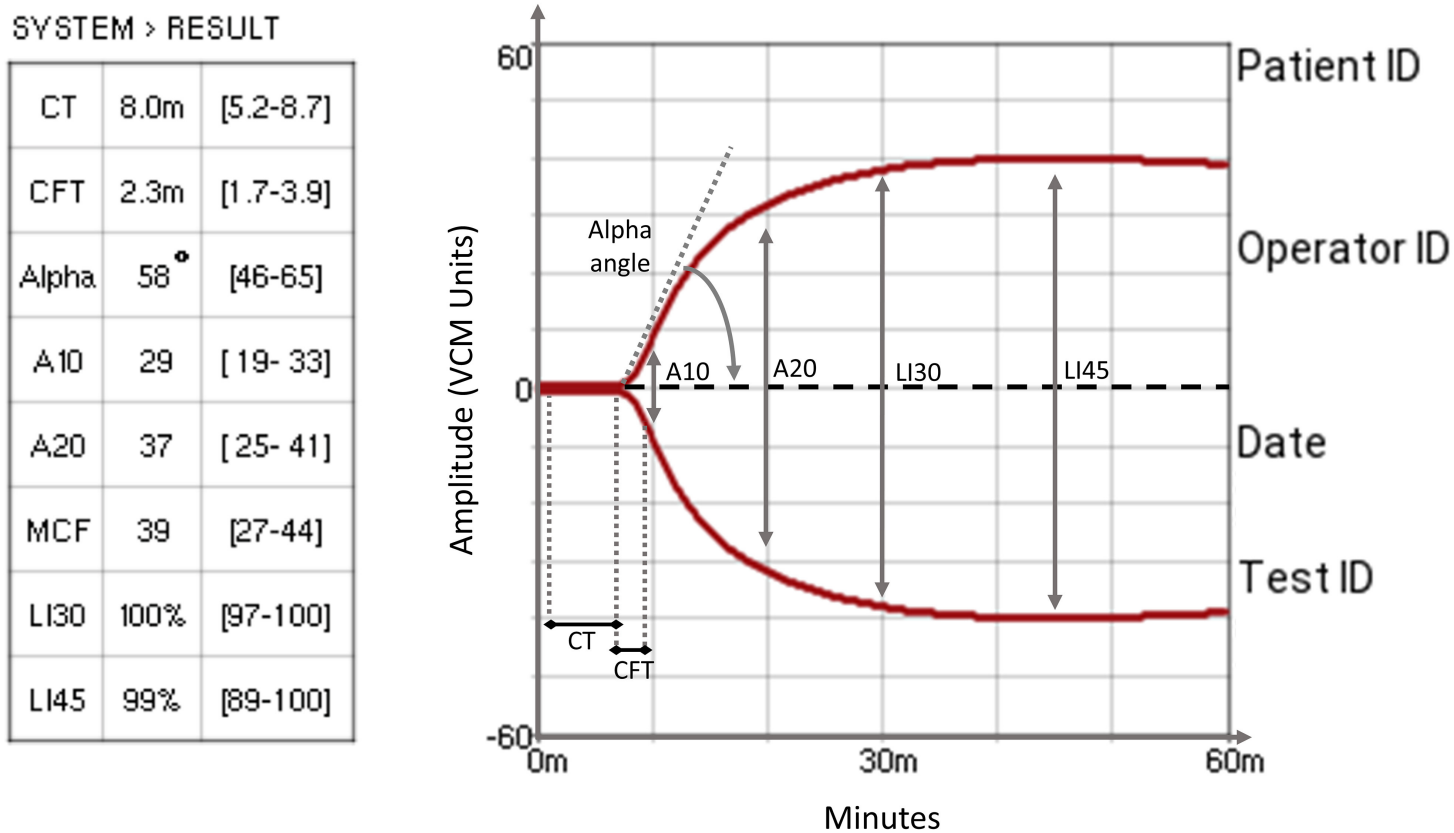
A typical VCM trace that shows VCM measurement parameters. *CT*, clotting time: the time required to reach the clot amplitude of 1% above the baseline; *CFT*, clot formation time: the time required to pass from 1 to 10% amplitude of the clot signal, describing the kinetics of the clot; *Alpha angle*: the angle between the baseline and the tangent to the curve at an amplitude of 1%, describing the kinetics of the clot; *A10 & A20*, amplitude at 10 and 20 min: clot amplitude measured at 10 and 20 min, describing clot firmness; *MCF*, maximum clot firmness: the maximum amplitude reached before the beginning of fibrinolysis, describing the firmness and quality of the clot; *LI30 & LI45*, lysis at 30 and 45 min: percentage clot amplitude at 30 and 45 min referred to MCF (14).

The VCM devices and cartridges were provided for no charge by the maker (Entegrion, Inc., Durham, NC, USA) to our Unit in 2019 to be used in NICU patients. The VCM display provides live numerical and graphical visualization of seven parameters: clotting time (CT), clot formation time (CFT), Alpha angle (Alpha), amplitude at 10 and 20 min (A10, A20), maximum clot firmness (MCF), lysis index at 30 and 45 min (LI30, LI45). Figure 1 shows a normal test and highlights the graphical representation of each parameter. The reference values displayed by the device refer to a population of 180 healthy adult volunteers (14). The technical properties and the description of the analytical procedure of VCM have been comprehensively discussed in a recent publication (15). Therefore, all seven parameters were included in the analysis.

### Statistical Analysis

Clinical and laboratory data were collected from the electronic patient medical charts, anonymized, and stored in a dedicated database. Data were analyzed using R statistical software, version 4.1.2 (R Foundation for Statistical Computing, Vienna, Austria). Descriptive statistics were presented for the overall population and grouped according to GA (<37 vs. ≥37 weeks of gestation) and clinical stability (patients requiring or not mechanical ventilation or vasopressor drugs). Continuous variables were described as mean (standard deviation) or median (interquartile range) depending on normality. The independent *t*-test and Mann-Whitney *U* test were used to compare VCM profiles, coagulation tests, and blood count between groups. The relationships between VCM parameters, coagulation tests, and blood count are presented using scatterplots. Considering that PT and aPTT showed a non-normal distribution, Spearman's rho coefficients were computed to study the correlation between CT and PT and CT and aPTT. Single linear regression models were used instead to study the association between (1) platelet (independent variable) and VCM parameters (dependent variables), (2) fibrinogen (independent variable), and VCM parameters. Aiming to compare VCM parameters' values in the infant population and the reference limits of the adult population, firstly, we computed the *t*-test between the means in the two groups. Moreover, we estimated the reference limits of our population as mean ± 1.96 SD and reported the proportion of neonates with VCM values falling outside the adult limits. All *p* < 0.05 were considered statistically significant.

## Results

**Figure 2:**
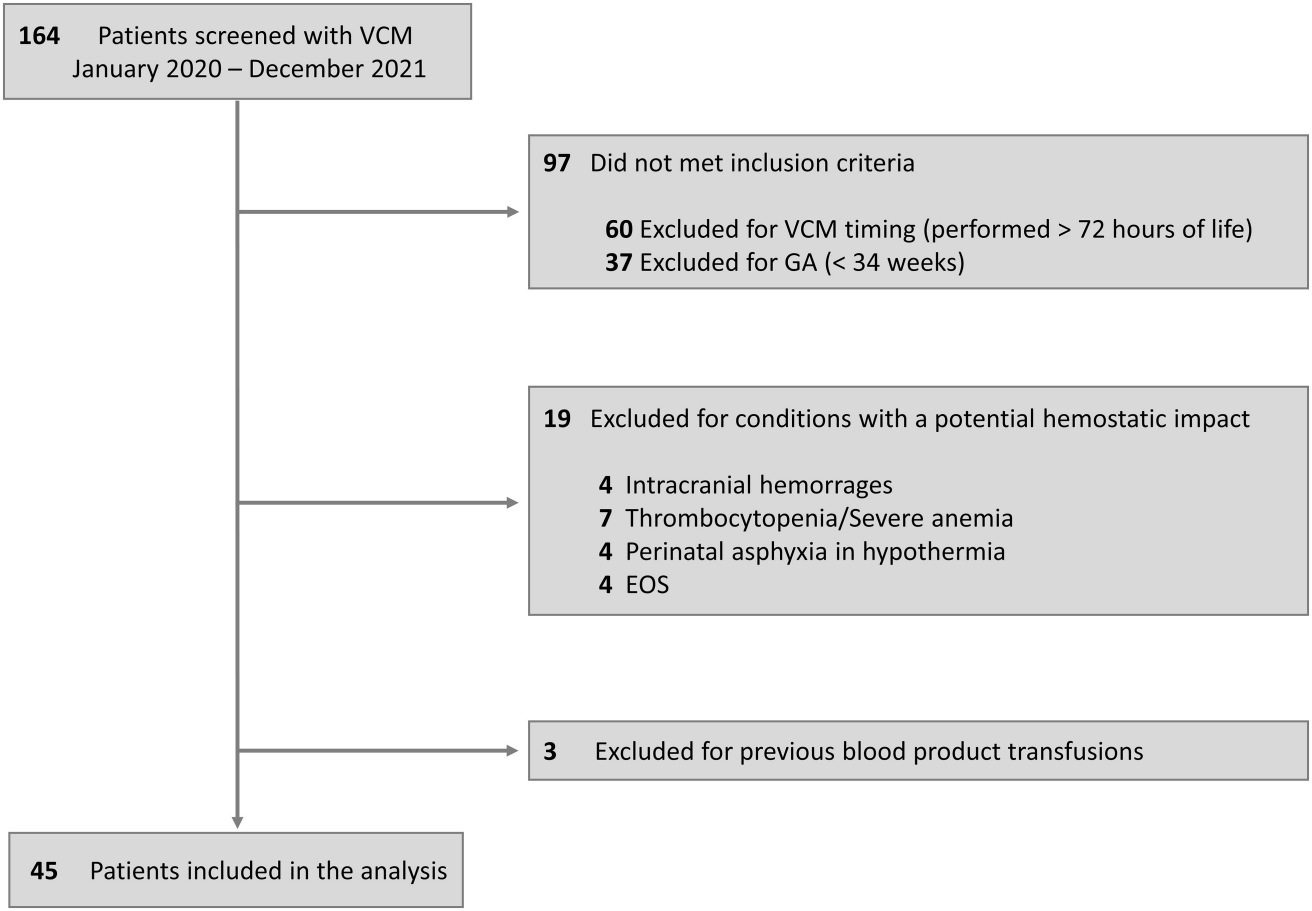
Flow diagram. VCM, viscoelastic coagulation monitor; GA, gestational age; EOS, early-onset sepsis.

We screened 164 neonates for 417 VCM assessments performed; of this cohort, 97 neonates did not meet the inclusion criteria, and 22 were excluded for conditions with a potential hemostatic impact or previous transfusions. Therefore, we enrolled 45 neonates, 26 FT, and 19 LP. The flow diagram is described in Figure 2. Demographic characteristics for the overall population are summarized in Table 1. Almost 90% of the cohort (40 out of 45) performed the VCM test as a pre-operative surgical evaluation, 21 (52.5%) in the elective regime and 19 (47.5%) in emergency conditions; 19 neonates (42.2%) required mechanical ventilation, of which 13 neonates (28.9%) required inotropes; all neonates had a balanced pH at the time of VCM test (Table 1).

**Table 1:** Characteristics of the study population.

**Baseline characteristics**	**Total (*N* = 45)**
GA (weeks), mean (SD)	37.1 (1.8)
*37+0 – 40+6, n (%)*	26 (57.8)
*34+0 – 36+6, n (%)*	19 (42.2)
Birth weight (g), mean (SD)	2,882 (665)
Cesarean section, n (%)	31 (68.9)
Male, n (%)	32 (71.1)
APGAR score 1', median (IQR)	8 (6–9)
APGAR score 5', median (IQR)	9 (8–10)
Mechanical ventilation, n (%)	19 (42.2)
Inotropes, n (%)	13 (28.9)
pH (venous), mean (SD)	7.34 (0.05)
**Admission diagnosis**	
**Surgical problems (pre-operative tests), n (%)**	40 (88.8)
Thoracic/Abdominal surgery, n (%)	31 (68.9)
Urological surgery, n (%)	4 (8.9)
Neurosurgery, n (%)	3 (6.7)
Cardiac surgery, n (%)	2 (4.4)
**Not surgical problems, n (%)**	5 (11.1)
Respiratory distress, n (%)	4 (8.9)
Jaundice (HDN excluded), n (%)	1 (2.2)
GA, gestational age; HDN, hemolytic disease of the newborns.

**Figure 3:**
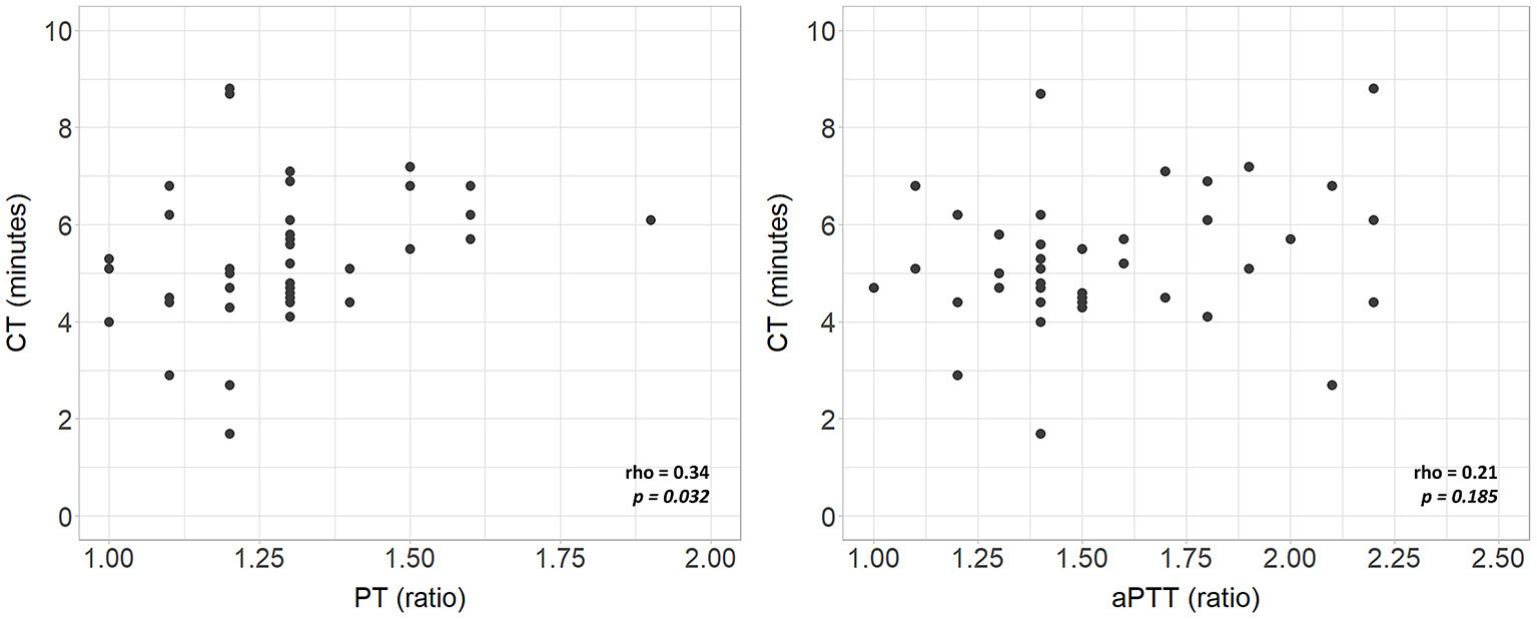
Scatterplots of VCM parameters and corresponding laboratory coagulation parameters: PT and aPTT. rho, Spearman's correlation coefficient; p, p-value; PT, prothrombin time; aPTT, activated partial thromboplastin time.

**Figure 4:**
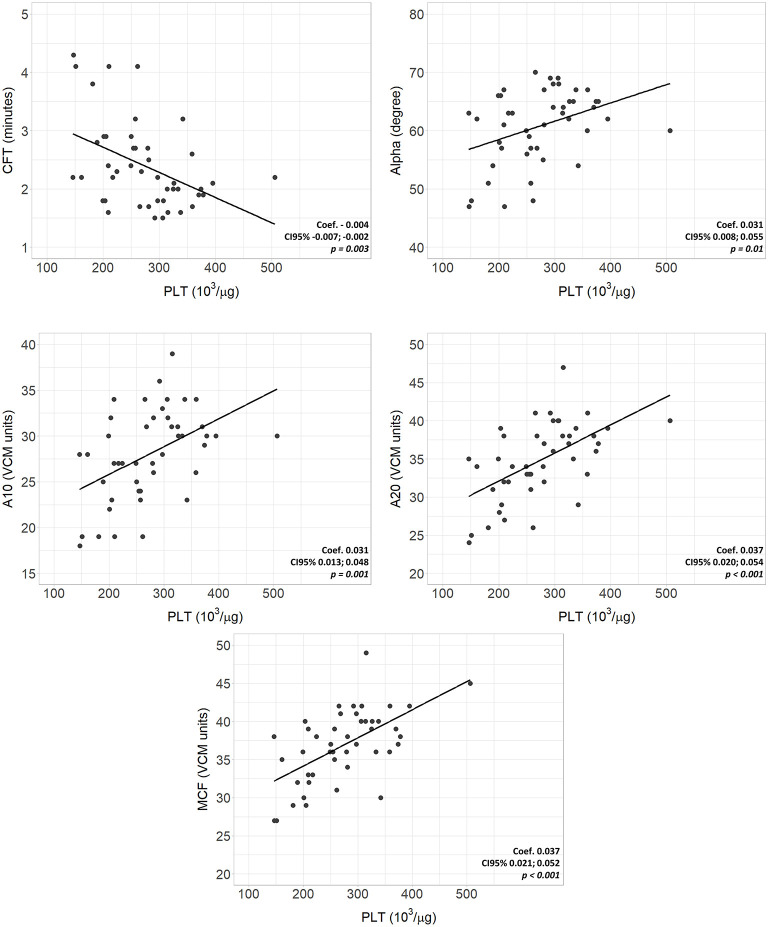
Scatterplots of VCM parameters and corresponding laboratory coagulation parameters: Platelets. Coef, linear regression coefficient; p, p-value; PLT, platelets; CFT, clotting formation time; Alpha, alpha angle; A10, amplitude at 10 min; A20 amplitude at 20 min; MCF, main clotting formation.

**Figure 5:**
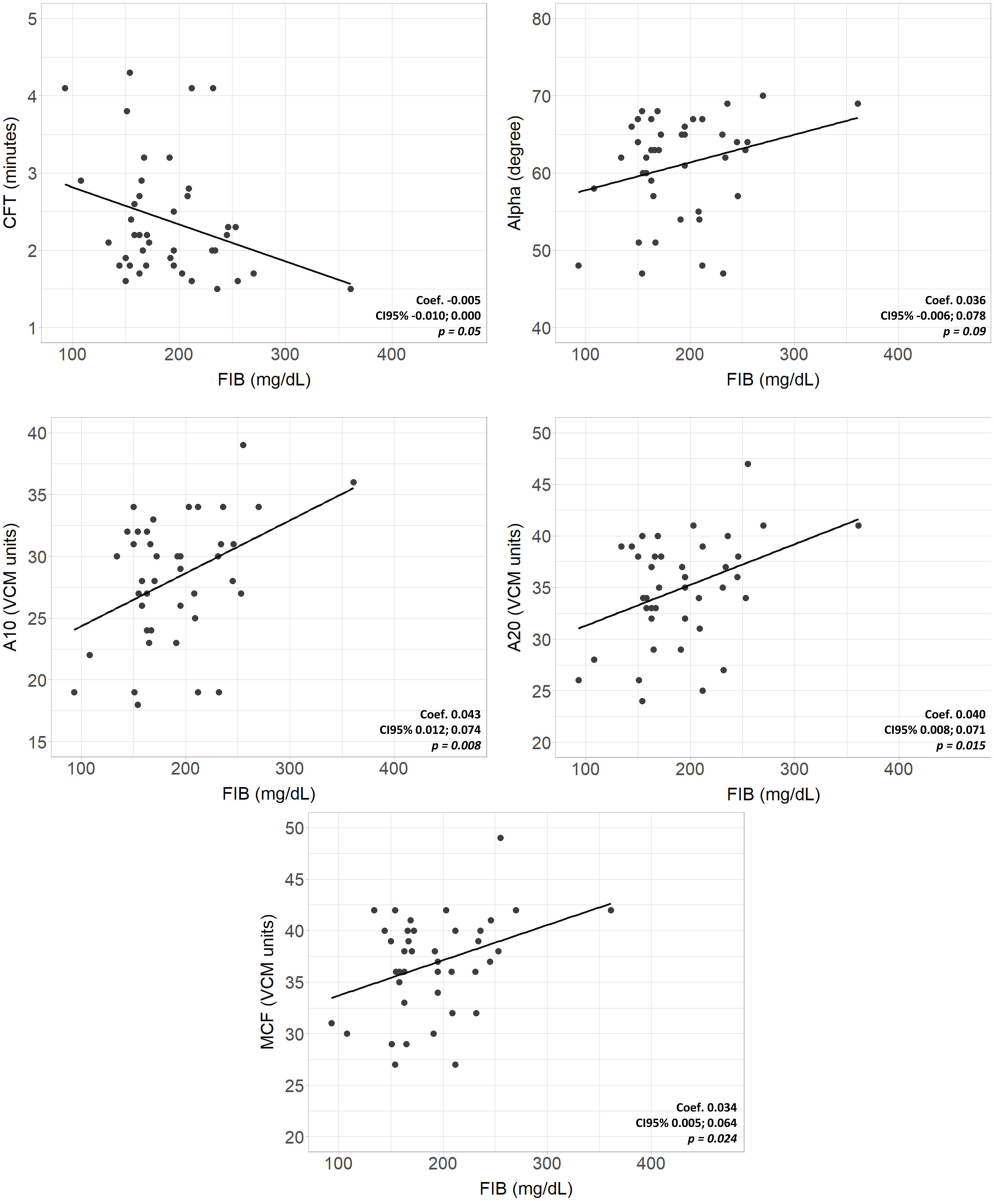
Scatterplots of VCM parameters and corresponding laboratory coagulation parameters: Fibrinogen. Coef, linear regression coefficient; p, p-value; FIB, fibrinogen; CFT, clotting formation time; Alpha, alpha angle; A10, amplitude at 10 min; A20 amplitude at 20 min; MCF, main clotting formation.

Complete VCM profile, coagulation tests, and blood count of the population are reported in Table 2. The population presented physiological hemoglobin, hematocrit, platelet, and fibrinogen values. PT and aPTT tests showed mean values of 1.29 and 1.57, respectively (physiological upper limit in adults 1.20). No significant differences in coagulation tests and blood count between subgroups were found. Scatterplots between standard laboratory parameters and VCM parameters are shown in Figure 3. CT was significantly correlated with PT (rho = 0.34, *p* = 0.032) but not with aPTT (rho = 0.21, *p* = 0.185). Platelet values resulted negative associated with CFT (*p* = 0.003) and positive related with Alpha (*p* = 0.010), A10 (*p* = 0.001), A20 (*p* < 0.001) and MCF (*p* < 0.001) (Figure 4). Fibrinogen showed a linear significant association with A10 (*p* = 0.001), A20 (*p* < 0.001), MCF (*p* < 0.001) (Figure 5).

**Table 2:** VCM profiles, coagulation tests, and blood count of the neonatal population.

**Variable**	**Total (*N* = 45)**	**FT (*N =* 26)**	**LP (*N =* 19)**	***p***	**Stable (*N =* 26)**	**Unstable (*N =* 19)**	***p***
CT (minutes)	5.3 (1.4)	5.0 (1.5)	5.7 (1.1)	0.098	5.3 (1.2)	5.4 (1.6)	0.768
CFT (minutes)	2.4 (0.7)	2.3 (0.7)	2.5 (0.8)	0.427	2.5 (0.8)	2.3 (0.7)	0.382
Alpha (degrees)	60.8 (6.3)	61.4 (6.3)	59.9 (6.7)	0.430	59.8 (6.6)	62.1 (5.8)	0.233
A10 (VCM units)	28.0 (5.0)	28.6 (4.7)	27.2 (5.3)	0.327	27.6 (5.1)	28.6 (4.8)	0.490
A20 (VCM units)	34.8 (5.0)	35.6 (4.7)	33.6 (5.3)	0.193	34.6 (5.2)	35.0 (4.8)	0.757
MCF (VCM units)	36.8 (4.8)	37.8 (4.2)	35.5 (5.3)	0.113	36.9 (5.0)	36.7 (4.5)	0.899
LI30 (%)	99.8 (0.5)	99.8 (0.5)	99.7 (0.4)	0.624	99.8 (0.4)	99.7 (0.6)	0.624
LI45 (%)	96.7 (2.4)	96.9 (2.3)	96.6 (2.6)	0.691	96.8 (2.8)	96.7 (2.0)	0.976
PT ratio	1.3 [1.2, 1.3]	1.3 [1.1, 1.3]	1.3 [1.2, 1.5]	0.123∧	1.3 [1.2, 1.4]	1.3 [1.2, 1.3]	0.607∧
aPTT ratio	1.5 [1.4, 1.8]	1.4 [1.4, 1.6]	1.6 [1.4, 1.9]	0.306∧	1.4 [1.4, 1.8]	1.5 [1.3, 1.8]	0.730∧
Fibrinogen (mg/dL)	190 (49)	205 (86)	190 (56)	0.972	193 (46)	185 (54)	0.621
Hb (g/dl)	15.7 (3.2)	15.9 (3.3)	15.6 (3.1)	0.837	16.3 (3.2)	14.8 (3.1)	0.123
Hct (%)	45.3 (9.5)	45.7 (9.9)	45.1 (9.1)	0.921	47.3 (9.7)	42.6 (8.7)	0.101
Platelet count (10^3^/μL)	273 (76)	281 (71)	270 (89)	0.802	279 (74)	265 (81)	0.570

*Values are expressed as mean (SD) or median [IQR]; FT, full-term infants; LP, late preterm infants; Stable, patients not requiring mechanical ventilation and vasopressor drugs; Unstable, patients requiring mechanical ventilation and vasopressor drugs. CT, clotting time; CFT, clotting formation time; Alpha, alpha angle; A10, amplitude at 10 min; A20 amplitude at 20 min; MCF, main clotting formation; LI30, lysis index at 30 min; LI45, lysis index at 45 min; PT, prothrombin time; aPTT, activated partial thromboplastin time; Hb, hemoglobin; Hct, hematocrit. p-values from t-test or from ∧Mann Whitney U test*.

In the first 72 h of life, neonates presented shorter CT and CFT, higher Alpha, A10, A20, and LI30 than healthy adults; MCF and LI45 were not different between the two populations (Table 3). The comparison between adults' reference limits and the ones computed on our population, along with the number of neonates falling below or above the adults' limits, is presented in Table 4.

**Table 3:** VCM profile, comparison between neonatal population and healthy adult population.

**VCM parameters**	**Neonatal population (*N* = 45)**		**Healthy adult population (*N* = 180)**		**Δ (%)**	p*
	**Mean**	**SD**	**Mean**	**SD**		
CT (minutes)	5.3	1.4	7.0	0.9	−1.7 (-24.3)	<0.001
CFT (minutes)	2.4	0.7	2.8	0.6	−0.4 (-14.3)	<0.001
ALPHA (degrees)	60.8	6.3	55	5	5.8 (10.5)	<0.001
A10 (VCM units)	28.0	5.0	26	4	2.0 (7.7)	0.004
A20 (VCM units)	34.8	5.0	33	4	1.8 (5.4)	0.012
MCF (VCM units)	36.8	4.8	36	4	0.8 (2.2)	0.225
LI30 (%)	99.8	0.5	99	1	0.8 (0.8)	<0.001
LI45 (%)	96.7	2.4	96	3	0.7 (0.7)	0.122

*Healthy adult population values have been provided by the manufacturer*;

**Δ**, *absolute (percentage) differences between neonatal cohort and healthy adult population*.

* *p-values from t-test*.

**Table 4:** VCM profiles, comparison between neonatal population and adult reference limits.

**VCM parameters**	**Healthy adult population**	**Neonatal population**			
	**Limits**	**Limits**	**N**	**Lower, n (%)**	**Higher, n (%)**
CT (minutes)	5.2–8.7	2.6–8.1	45	23 (51.1)	1 (2.2)
CFT (minutes)	1.7–3.9	0.9–3.9	45	5 (11.1)	4 (8.9)
ALPHA (degrees)	46–65	48.4–73.1	45	0 (0.0)	11 (24.4)
A10 (VCM units)	19–33	18.2–37.8	45	1 (2.2)	7 (15.6)
A20 (VCM units)	25–41	25.0–44.6	45	1 (2.2)	1 (2.2)
MCF (VCM units)	27–44	27.4–46.2	45	0 (0.0)	2 (4.4)
LI30 (%)	97–100	98.8–100.0	45	0 (0.0)	0 (0.0)
LI45 (%)	89–100	92.0–100.0	45	0 (0.0)	0 (0.0)

*Reference limits values are calculated as mean ±1.96 SD [the manufacturer's manual provides adult reference limits (8)]; Lower, infants with parameter values below the lower adult reference population limit; Higher, infants with parameter values above the upper adult reference population limit*.

## Discussion

This study provides a viscoelastic profile performed with the VCM device in a cohort of non-coagulopathic FT and LP infants during the first 72 h of life. The population studied was hemostatically homogeneous as no differences in VCM parameters were found analyzing subgroups according to GA and clinical conditions. The relationship between standard hemostatic tests and the clotting time was poor, especially for aPTT (16). Despite prolonged aPTT, compared to adults, neonates need shorter times to start the clot, with more than 50% of neonates showing a CT below the lower reference limit. Accordingly, the correlation between PT and CT is not clinically usable since prolonged PT values correspond to standard or short coagulation times, as shown in Figure 3.

On the other hand, platelet values showed a significant association with CFT, Alpha, and amplitude parameters, accelerating the clot formation and, together with fibrinogen, increasing the strength of the clot. Moreover, with platelet and fibrinogen values within the physiological limits, neonates show shorter times than adults to reach a significant clot, with nearly 25% of neonates with an alpha angle above the upper reference limit. This suggests a qualitative difference between these two populations, confirming the comprehensive and not just quantitative assessment provided by viscoelastic tests. Therefore, the neonatal viscoelastic profile maintains a hemostatic competence, which is confirmed by the bleeding-free clinical course of the patients. Previous viscoelastic studies on newborns using TEG and ROTEM systems confirm our results, showing accelerated coagulation and clot formation time in healthy term newborns compared to children and adults (17–19). These findings reflect the “developmental hemostasis”, defined as the physiological ontogenetic process that leads to a mature coagulation system (20, 21). Hemostatic competence is a physiologically balanced process at all ages, but with dynamic changes in the plasma concentration of the factors involved. The coagulation system forms early during fetal growth; nevertheless, preterm infants have lower levels of pro-coagulant and anti-coagulant factors than term newborns. Their synthesis increases proportionally after the 34th week of gestation, but most do not reach adult values at birth (12). The significant vitamin K-dependent factors and contact factor deficiencies lead to the misleading alteration of PT and aPTT (22). Indeed, traditional coagulation tests neglect the simultaneous deficit of coagulation inhibitors and, consequently, have a poor correlation with the *in vivo* coagulation time evaluated through a viscoelastic test. Conversely, factors V, VIII, von Willebrand, platelets, and fibrinogen are already comparable to adult levels at birth, explaining the pro-coagulant CT, CFT, and Alpha (23).

Other minor differences in the parameters that reflect clot strength and clot lysis (A10, A30, and LI30) have an uncertain biological significance. Compared to adults, our patients show only temporary changes in amplitude and fibrinolysis, as the late parameters of clot strength and fibrinolysis (MCF and LI45), which are more relevant, show no differences between the two populations. The neonatal characteristics of transient platelet hyporeactivity, impaired fibrin polymerization, the reduction of tissues plasminogen activator, and increased plasminogen activator inhibitor may suggest poorer clot strength and lower fibrinolytic activity; the literature data are conflicting and require further investigations (12, 23–25).

Hence, the neonatal hemostatic profile displayed by VMC presents characteristics comparable to traditional viscoelastic tests despite having different analytical procedures and application profiles.

TEG5000® and Rotem® technology analyze the variations of a rotational force applied to an electromechanical transducer suspended in the blood sample (26). Differently, VCM detects, through an optical sensor, the variations of reciprocal movement between two surfaces of a glass capillary induced by the coagulation of the interposed blood. This method allows the use of untreated whole blood, exploiting the hemostatic activation induced by the glass surface. In addition, eliminating the activator or additive decreases the risk of pre-analytical error and reduces the blood volume needed (<500 μL).

However, these characteristics also involve some limitations. Firstly, the requirement to perform the analysis within 4 min from the blood collection. Moreover, the impossibility of evaluating the hemostatic profile with heparin neutralizers or with antifibrinolytic drugs (14). This implies, for example, the impossibility of using the VCM for the coagulation monitoring of a patient in extracorporeal membrane oxygenation (ECMO) support and, in general, to monitor the therapeutic contribution to hemostasis in anticoagulated patients. Specific thromboelastographic systems also allow coagulation assays that highlight the contribution of the different coagulation pathways, such as the ClotPro® analyzer. The main advantage of VCM, which makes it attractive for neonatology, is that it is a portable cordless device, convenient for quick and bedside analysis immediately after sampling in NICU, delivery room, or emergency departments. Of note, its compactness prevents simultaneous analysis of multiple samples.

This is the first study reporting VCM parameters in a non-coagulopathic cohort of neonates, and the results, in line with the evidence from other viscoelastic techniques, support its use in a NICU setting. However, the study's limitations that need to be considered are the size of the population, insufficient to provide proper reference intervals and its clinical heterogeneity. Inflammation affects hemostatic properties, activating a more pro-coagulant endothelial phenotype. Conversely, conditions such as respiratory distress syndrome (RDS) can induce a hypocoagulable profile (27, 28). Consequently, the standard viscoelastic profile should ideally be studied in a population without acute conditions. Neonates with elective surgical problems, which represent about 50% of our population, are the best compromise for viscoelastic study in a NICU setting, as they are clotting-competent patients routinely subjected to hemostatic evaluation. Another limitation was the inability to retrospectively establish the origin of the sampling (venous/arterial, peripheral/central). However, a recent trial on newborns evaluated with VCM has shown no significant inter-individual differences by varying the method and the blood collection site (29).

## Conclusion

In conclusion, VCM represents a rapid and easy-to-use resource for bedside assessment of coagulopathy in NICU, useful in association with another viscoelastic device capable of simultaneously analyzing the hemostatic contribution of heparin therapy. VCM parameters in FT and LP infants appear to be comparable to the adult population's reference intervals, except for the clotting time, clotting formation time, and alpha angle, which are slightly shorter and higher, respectively, in newborns. This may suggest that longer coagulation times should be more alarming than pro-coagulant values in neonates without prothrombotic risk factors. Further clinical studies on healthy term and preterms are needed to diversify the reference intervals of VCM in the neonatal population and, eventually, to include this novel tool in neonatal transfusion algorithms.

## Data Availability Statement

The raw data supporting the conclusions of this article will be made available by the authors, without undue reservation.

## Ethics Statement

The studies involving human participants were reviewed and approved by Milan Area 2, Italy. Written informed consent from the participants' legal guardian/next of kin was not required to participate in this study in accordance with the national legislation and the institutional requirements.

## Author Contributions

GA, SGh, GR, IA, SGu, VC, FMa, and GC contributed to the study's conception and design. GA, SGh, GR, IA, SGu, VC, FMa, and GC wrote the first draft of the manuscript. GA and NP performed the statistical analysis. GA, IA, SGu, VC, and FMa assessed the viscoelastic coagulation tests. SGh, GR, IA, GC, and FMo provided extensive critical revision. All authors contributed to the manuscript's critical revision and read and approved the submitted version.

## Funding

This study was (partially) supported by the Italian Ministry of Health (Ricerca Corrente 2022).

## Conflict of Interest

The authors declare that the research was conducted in the absence of any commercial or financial relationships that could be construed as a potential conflict of interest.

## Publisher's Note

All claims expressed in this article are solely those of the authors and do not necessarily represent those of their affiliated organizations, or those of the publisher, the editors and the reviewers. Any product that may be evaluated in this article, or claim that may be made by its manufacturer, is not guaranteed or endorsed by the publisher.

## References

[1] Sewell EK Forman KR Wong EC Gallagher M Luban NL Massaro AN. Thromboelastography in term neonates: an alternative approach to evaluating coagulopathy. Arch Dis Child Fetal Neonatal Ed. (2017) 102:F79–84. 10.1136/archdischild-2016-310545 27178714

[2] Davenport P Sola-Visner M. Hemostatic challenges in neonates. Front Pediatr. (2021) 9:627715. 10.3389/fped.2021.627715 33738269 PMC7960661

[3] Parastatidou S Sokou R Tsantes AG Konstantinidi A Lampridou M Ioakeimidis G. The role of ROTEM variables based on clot elasticity and platelet component in predicting bleeding risk in thrombocytopenic critically ill neonates. Eur J Haematol. (2021) 106:175–83. 10.1111/ejh.13534 33053216

[4] Radicioni M Bruni A Bini V Villa A Ferri C. Thromboelastographic profiles of the premature infants with and without intracranial hemorrhage at birth: a pilot study. J Matern Fetal Neonatal Med. (2015) 28:1779–83. 10.3109/14767058.2014.968773 25245227

[5] Sokou R Piovani D Konstantinidi A Tsantes AG Parastatidou S Lampridou M. A risk score for predicting the incidence of hemorrhage in critically ill neonates: development and validation study. Thromb Haemost. (2021) 121:131–9. 10.1055/s-0040-1715832 32838471

[6] Sokou R Tsantes AG Konstantinidi A Ioakeimidis G Lampridou M Parastatidou S. Rotational thromboelastometry in neonates admitted to a neonatal intensive care unit: a large cross-sectional study. Semin Thromb Hemost. (2021) 47:875–84. 10.1055/s-0041-1729964 34130345

[7] Sokou R Tritzali M Piovani D Konstantinidi A Tsantes AG Ioakeimidis G. Comparative performance of four established neonatal disease scoring systems in predicting in-hospital mortality and the potential role of thromboelastometry. Diagnostics (Basel). (2021) 11. 10.3390/diagnostics11111955 34829302 PMC8619208

[8] Monagle P Ignjatovic V Savoia H. Hemostasis in neonates and children: pitfalls and dilemmas. Blood Rev. (2010) 24:63–8. 10.1016/j.blre.2009.12.001 20074839

[9] Tripodi A Chantarangkul V Mannucci PM. Acquired coagulation disorders: revisited using global coagulation/anticoagulation testing. Br J Haematol. (2009) 147:77–82. 10.1111/j.1365-2141.2009.07833.x 19659548

[10] Christensen RD Baer VL Lambert DK Henry E Ilstrup SJ Bennett ST. Reference intervals for common coagulation tests of preterm infants (CME). Transfusion. (2014) 54:627–32:quiz 6. 10.1111/trf.12322 23834237

[11] Tripodi A Ramenghi LA Chantarangkul V De Carli A Clerici M Groppo M. Normal thrombin generation in neonates in spite of prolonged conventional coagulation tests. Haematologica. (2008) 93:1256–9. 10.3324/haematol.12566 18403390

[12] Jaffray J Young G. Developmental hemostasis: clinical implications from the fetus to the adolescent. Pediatr Clin North Am. (2013) 60:1407–17. 10.1016/j.pcl.2013.08.003 24237979

[13] Raffaeli G Tripodi A Cavallaro G Cortesi V Scalambrino E Pesenti N. Thromboelastographic profiles of healthy very low birthweight infants serially during their first month. Arch Dis Child Fetal Neonatal Ed. (2020) 105:412–8. 10.1136/archdischild-2019-317860 31704736

[14] Entegrion. VCM Operators Manual. For the near-patient semi-quantitative viscoelastic measurement of coagulation in whole blood. Durham, NC: Entegrion, Inc., (2018).

[15] Panigada M Meli A Scotti E Properzi P Brioni M Kamel S. Viscoelastic coagulation monitor as a novel device to assess coagulation at the bedside. A single-center experience during the COVID-19 pandemic. ASAIO J. (2021) 67:254–62. 10.1097/MAT.0000000000001380 33627598

[16] Haas T Spielmann N Mauch J Madjdpour C Speer O Schmugge M. Comparison of thromboelastometry (ROTEM(R)) with standard plasmatic coagulation testing in paediatric surgery. Br J Anaesth. (2012) 108:36–41. 10.1093/bja/aer342 22086509

[17] Edwards RM Naik-Mathuria BJ Gay AN Olutoye OO Teruya J. Parameters of thromboelastography in healthy newborns. Am J Clin Pathol. (2008) 130:99–102. 10.1309/LABNMY41RUD099J2 18550478

[18] Strauss T Levy-Shraga Y Ravid B Schushan-Eisen I Maayan-Metzger A Kuint J. Clot formation of neonates tested by thromboelastography correlates with gestational age. Thromb Haemost. (2010) 103:344–50. 10.1160/TH09-05-0282 20076842

[19] Oswald E Stalzer B Heitz E Weiss M Schmugge M Strasak A. Thromboelastometry (ROTEM) in children: age-related reference ranges and correlations with standard coagulation tests. Br J Anaesth. (2010) 105:827–35. 10.1093/bja/aeq258 20884636

[20] Andrew M Paes B Milner R Johnston M Mitchell L Tollefsen DM. Development of the human coagulation system in the full-term infant. Blood. (1987) 70:165–72. 10.1182/blood.V70.1.165.165 3593964

[21] Andrew M Paes B Milner R Johnston M Mitchell L Tollefsen DM. Development of the human coagulation system in the healthy premature infant. Blood. (1988) 72:1651–7. 10.1182/blood.V72.5.1651.1651 3179444

[22] Schott NJ Emery SP Garbee C Waters J. Thromboelastography in term neonates. J Matern Fetal Neonatal Med. (2018) 31:2599–604. 10.1080/14767058.2017.1349747 28662614

[23] Katsaras G Sokou R Tsantes AG Piovani D Bonovas S Konstantinidi A. The use of thromboelastography (TEG) and rotational thromboelastometry (ROTEM) in neonates: a systematic review. Eur J Pediatr. (2021) 180:3455–70. 10.1007/s00431-021-04154-4 34131816

[24] Ferrer-Marin F Sola-Visner M. Neonatal platelet physiology and implications for transfusion. Platelets. (2022) 33:14–22. 10.1080/09537104.2021.1962837 34392772 PMC8795471

[25] Cvirn G Gallistl S Kutschera J Wagner T Ferstl U Jurgens G. Clot strength: a comparison between cord and adult blood by means of thrombelastometry. J Pediatr Hematol Oncol. (2008) 30:210–3. 10.1097/MPH.0b013e318162bd2c 18376283

[26] Whiting D DiNardo JA. TEG and ROTEM: technology and clinical applications. Am J Hematol. (2014) 89:228–32. 10.1002/ajh.23599 24123050

[27] Esmon CT. The interactions between inflammation and coagulation. Br J Haematol. (2005) 131:417–30. 10.1111/j.1365-2141.2005.05753.x 16281932

[28] Katsaras GN Sokou R Tsantes AG Konstantinidi A Gialamprinou D Piovani D. Thromboelastometry in neonates with respiratory distress syndrome: a pilot study. Diagnostics (Basel). (2021) 11. 10.3390/diagnostics11111995 34829342 PMC8618420

[29] Radicioni M Massetti V Bini V Troiani S. Impact of blood sampling technique on reproducibility of viscoelastic coagulation monitor (VCM) system test results in the neonate. J Matern Fetal Neonatal Med. (2021) 1–7. 10.1080/14767058.2021.1933935 34304670

